# Bilateral extensive adult-onset limbal xanthogranuloma invading the deep corneal stroma: a case report and literature review

**DOI:** 10.3389/fmed.2026.1856414

**Published:** 2026-06-12

**Authors:** Yuanyuan Tu, Yake Sun, Manhui Zhu, Qingliang Zhao

**Affiliations:** Department of Ophthalmology, Lixiang Eye Hospital of Soochow University, Suzhou, Jiangsu, China

**Keywords:** adult-onset xanthogranuloma, anterior segment optical coherence tomography, case report, lamellar keratoplasty, non-Langerhans cell histiocytosis

## Abstract

**Background:**

Adult-onset xanthogranuloma (AOXG) is a rare, benign form of non-Langerhans cell histiocytosis. Ocular manifestations commonly involve the orbit, uveal tract, or adnexal tissues; however, concurrent bilateral involvement accompanied by extensive infiltration of the corneoscleral limbus remains exceptionally rare and poses substantial diagnostic and therapeutic challenges.

**Case presentation:**

A 48-years-old male presented with a 20-years history of progressively enlarging bilateral, yellow-white corneoscleral lesions associated with severe visual impairment (right eye best-corrected visual acuity: 0.02). The patient subsequently underwent right eye corneal lesion excision combined with lamellar keratoplasty (LKP). Histopathological and immunohistochemical evaluation demonstrated a dense subepithelial infiltration of lipid-laden histiocytes and characteristic Touton giant cells, with immunoreactivity for CD68 and negative staining for S-100 and CD1a. Owing to the highly unusual bilateral presentation, a thorough systemic evaluation was initiated to exclude potentially lethal systemic histiocytoses, particularly Erdheim-Chester disease (ECD). Whole-body imaging revealed no evidence of visceral involvement or osteosclerotic lesions. Furthermore, molecular analysis confirmed a wild-type BRAF gene, while serum immunoelectrophoresis demonstrated no evidence of monoclonal gammopathy. Based on these comprehensive findings, a definitive diagnosis of AOXG was established. Following treatment, the patient demonstrated excellent anatomical resolution without evidence of recurrence and accompanied by a modest visual improvement to a BCVA of 0.1 throughout the 18-months follow-up period.

**Conclusion:**

Bilateral extensive limbal AOXG is a rare entity that can easily be misdiagnosed as corneal degeneration, squamous cell carcinoma, or sclerocornea. Persistent chronic inflammation alongside hyperlipidemia may have contributed to the disease’s etiology. Regarding lesions exhibiting deep stromal invasion, integrating LKP with intralesional corticosteroid administration may be a useful therapeutic approach in selected vision-threatening cases.

## Introduction

Adult-onset xanthogranuloma (AOXG) is classified within the clinical spectrum of juvenile xanthogranuloma (JXG)-related disorders and represents a subtype of non-Langerhans cell histiocytosis (1). Although JXG predominantly affects infants and young children, AOXG emerges in adulthood and typically manifests as solitary or multiple yellowish cutaneous nodules. Within the JXG spectrum, ocular involvement most commonly affects the iris, frequently resulting in spontaneous hyphema, or the conjunctiva (2). Isolated, bilateral involvement of the corneoscleral limbus is exceedingly rare, as evidenced by the scarce number of cases documented in the literature. Consequently, these cases are often misdiagnosed as other ocular surface neoplasms or degenerations (3).

The pathogenesis of epibulbar xanthogranuloma remains elusive. Current evidence suggests that its development may involve the interplay of multiple factors, including viral infections, localized tissue injury, and immune dysregulation (4–7). In this report, we present an exceptionally rare case of AOXG in a 48-years-old male patient presenting with a two-decade history of progressive disease, notably featuring infiltration into the deep corneal stroma. The condition was effectively managed via lamellar keratoplasty (LKP) in conjunction with adjunctive corticosteroid therapy.

This report describes an unusual case of massive, bilateral limbal AOXG with deep stromal invasion. Additionally, we outline a structured diagnostic approach and provide a comprehensive literature review to contextualize this rare condition.

## Case description

A 48-years-old Chinese male farmer presented to the ophthalmology department with bilateral, progressively enlarging yellow-white corneal lesions associated with severe visual impairment, with disease progression spanning the preceding 20 years. The patient reported a history of metallic foreign body penetration in both eyes occurring 20 years earlier. He had previously undergone partial excision at another hospital, with histopathological findings reported as “benign” without further specification. Notably, the lesions had demonstrated marked enlargement over the preceding 2 years. One month prior to admission, a superficial biopsy performed in the outpatient clinic indicated “chronic inflammation with fibrous tissue hyperplasia,” which demonstrated no clinical response to topical corticosteroids or antibiotics. Systemic history revealed hyperlipidemia (total cholesterol: 6.28 mmol/L; LDL: 4.15 mmol/L), with no history of hypertension, diabetes mellitus, or systemic autoimmune diseases.

**FIGURE 1:**
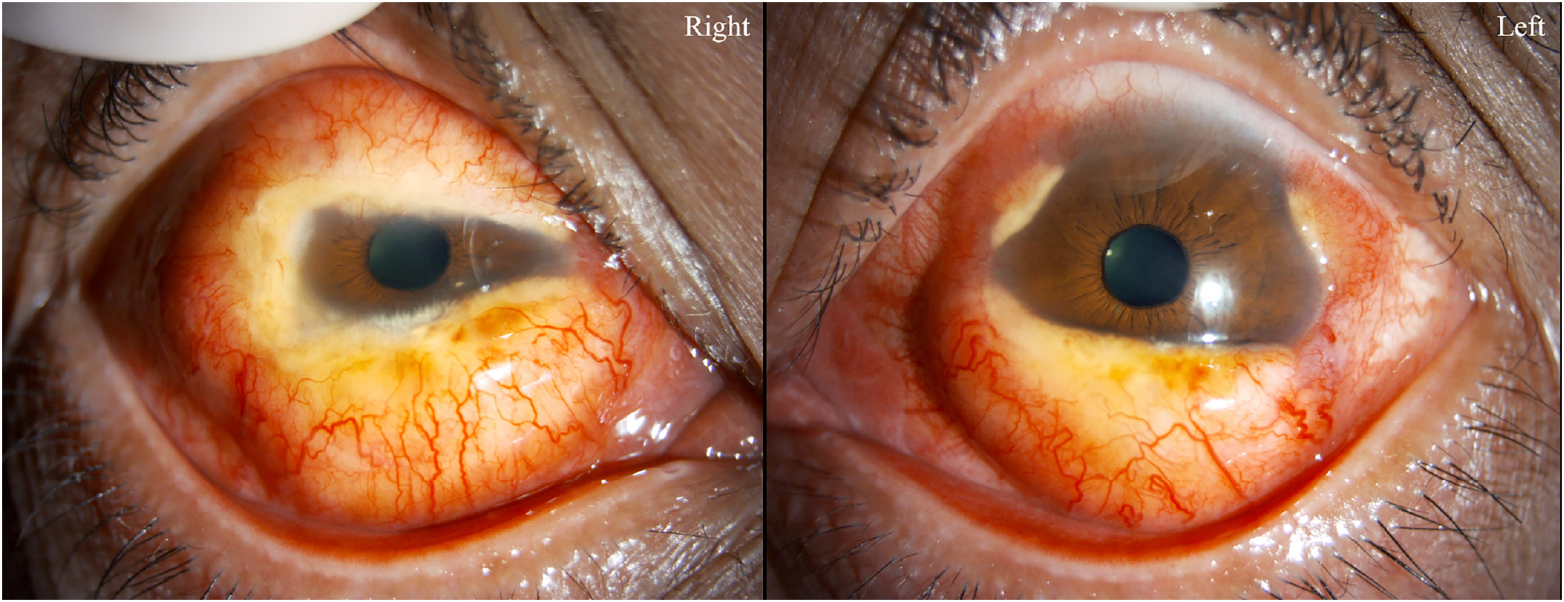
The slit-lamp photograph of the right **(A)** and left **(B)** eyes showing multiple yellowish masses situated at the corneoscleral region. These masses occupy the central corneal area and partially block the visual axis, with prominent blood vessels.

**FIGURE 2:**
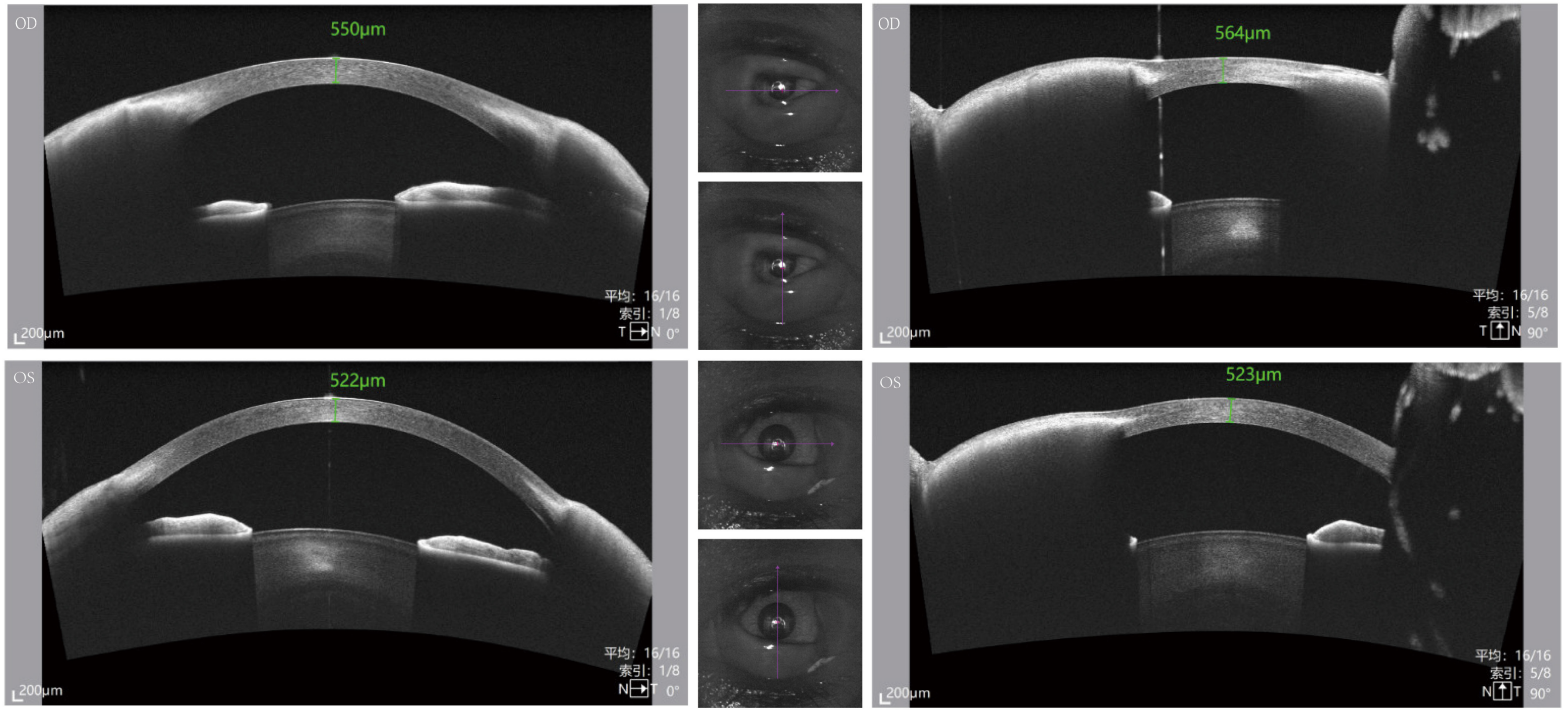
AS-OCT revealed irregular corneal thickening with the lesions invading the deep stroma.

Ophthalmic evaluation demonstrated a best-corrected visual acuity (BCVA) of 0.02 in the right eye (OD) and 0.4 (improving to 0.6 with correction) in the left eye (OS). Intraocular pressure (IOP) measurements were 10 mmHg OD and 17 mmHg OS. Slit-lamp biomicroscopic examination of the right eye disclosed marked conjunctival hyperemia alongside an extensive, 360-degree yellow-white xanthogranulomatous lesion characterized by prominent vascularization. This lesion originated 4–5 mm posterior to the limbus, extended into the central cornea, and partially obscured the visual axis. The left eye exhibited a similar, albeit less severe, vascularized lesion extending from the 2 to 11 o’clock positions, with more pronounced involvement inferiorly (Figure 1). AS-OCT revealed irregular corneal thickening, with lesional infiltration extending into the deep stromal layers (Figure 2). Complementary ultrasound biomicroscopy (UBM) imaging detected well-circumscribed hypoechoic masses localized to the corneal surface, while confirming that the anterior chamber angles remained open and structurally normal. Furthermore, *in vivo* confocal microscopy revealed epithelial surface irregularities, significant infiltration by inflammatory cells, and a marked reduction in subepithelial nerve fiber density.

The presence of deep stromal invasion on AS-OCT directly precluded simple excision due to the high risk of corneal perforation, and thereby necessitated LKP. Under general anesthesia, the patient underwent right eye corneal lesion excision. Intraoperative findings confirmed extension of the lesion into the deep stromal layers as well as the peripheral sclera. To restore corneal integrity and transparency, an anterior LKP was performed using a 13 mm donor graft. The choice of a 13-mm graft was selected to ensure complete coverage of extensive corneal and limbal stromal defects and while guaranteeing a clear visual axis. The depth of the lamellar dissection was guided by the posterior boundary delineated preoperatively using AS-OCT. As complete excision of the scleral component of the lesion was not feasible without compromising the globe integrity, an intralesional injection of triamcinolone acetonide (20 mg/0.5 ml) was administered into the residual scleral bed.

**FIGURE 3:**
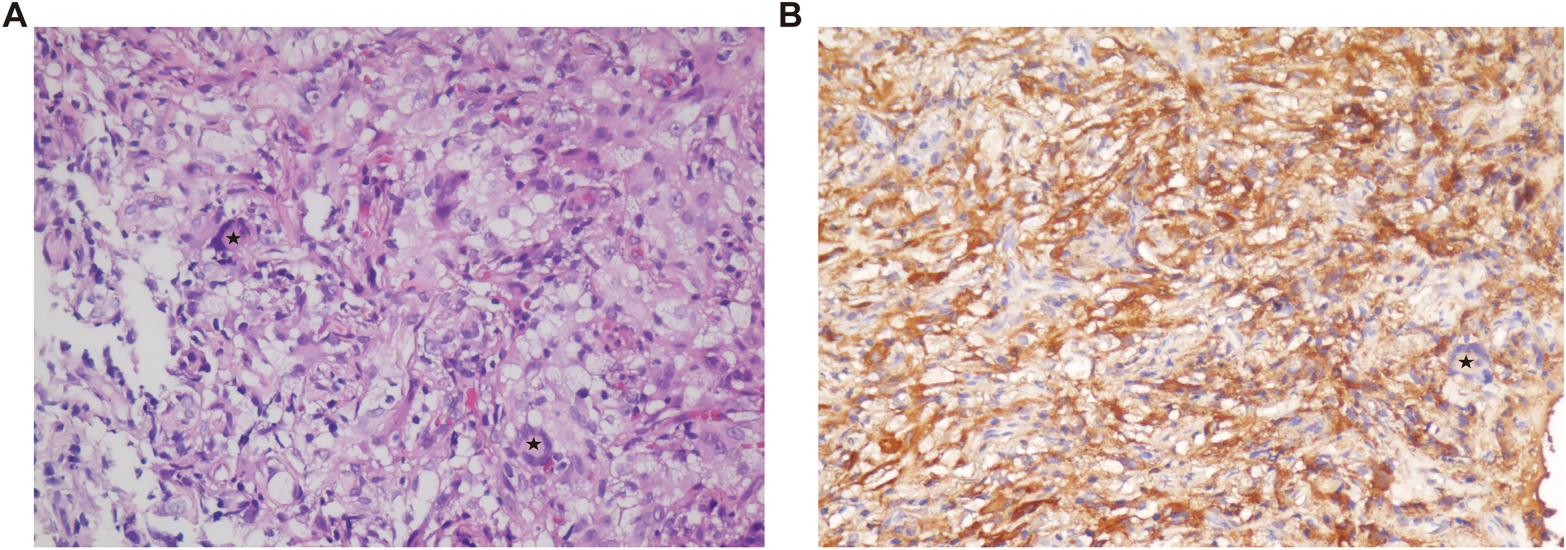
**(A)** Histopathology revealed chronic inflammation, fibrosis, and Touton giant cells. H&E stain, original magnification, ×200. **(B)** Immunohistochemistry reveals abundant macrophage aggregates in the corneal stroma, which are visualized by CD68 staining. *Examples of Touton’s giant cells; note the typical foamy xanthomatous cytoplasm peripheral to the ring of nuclei, original magnification, ×200.

Postoperatively, the patient developed a minor interface hemorrhage on day 2, which was successfully managed with anterior chamber/interface irrigation on day 3. Histopathological examination (hematoxylin and eosin staining, H&E) of the excised tissue revealed subepithelial fibrous tissue proliferation with extensive infiltration by histiocyte-like cells, foamy histiocytes, and scattered multinucleated giant cells exhibiting a characteristic wreath-like nuclear arrangement surrounded by foamy cytoplasm (classic Touton giant cells), accompanied by lymphocytic infiltration. Importantly, no evidence of collagen necrobiosis or atypical mitotic activity was identified. To accurately characterize and classify the histiocytic proliferation, an extensive immunohistochemical (IHC) panel together with molecular pathological analysis was subsequently performed. IHC staining revealed diffuse and strong positivity for the macrophage markers CD68 and CD163 in the lesional histiocytes and Touton giant cells In contrast, the cells demonstrated complete negativity for Langerhans-cell- associated markers, including CD1a, Langerin (CD207), and S-100 protein (Figure 3). Furthermore, molecular pathological analysis performed using polymerase chain reaction (PCR) confirmed a wild-type BRAF gene status with no evidence of the BRAF V600E mutation. Systemic evaluation demonstrated no history of asthma or lymph node disease, serum immunoglobulin levels (IgG4) were within the normal range, and radiographic examination revealed no abnormalities of the long bones. Collectively, the local immunomolecular pathological findings together with the negative systemic evaluation conclusively established the diagnosis of bilateral AOXG (Table 1).

**TABLE 1:** Summary of diagnostic workup and differential diagnosis.

Diagnostic category	Investigation/marker	Result in our patient	Clinical significance/excluded entity
Clinical and systemic workup	History of asthma/lymphadenopathy	Negative	Argues against AAPOX
	Serum immunofixation electrophoresis	Normal (no paraproteinemia)	Argues against NXG and Multiple Myeloma
	Serum IgG4 levels	Normal	Does not support IgG4-related ophthalmic disease
	Systemic lipid profile	Elevated (TC: 6.28 mmol/L; LDL: 4.15 mmol/L)	Suggests a predisposing metabolic factor
Systemic imaging	Long-bone plain radiography	Normal (no osteosclerosis)	Argues against ECD
Histopathology (H&E)	Morphological features	Foamy histiocytes, classic Touton giant cells present	Confirms xanthogranulomatous disease (JXG family)
	Collagen necrobiosis	Absent	Argues against NXG
	Cellular atypia and mitotic figures	Absent	Argues against OSSN and other malignancies
Immunohistochemistry	Macrophage markers (CD68, CD163)	Diffusely and strongly positive	Confirms non-LCH histiocyte lineage
	Langerhans cell markers (CD1a, Langerin, S-100)	Negative	Argues against LCH
	Melanocytic markers (Melan-A, HMB-45)	Negative	Argues against amelanotic conjunctival melanoma
Molecular pathology	BRAF V600E mutation analysis (via PCR)	Negative (wild-type)	Does not support classical mutation-driven ECD and LCH

AAPOX, adult-onset asthma and periocular xanthogranuloma; NXG, necrobiotic xanthogranuloma; ECD, Erdheim-Chester disease; JXG, juvenile xanthogranuloma; OSSN, ocular surface squamous neoplasia; LCH, Langerhans cell histiocytosis; TC, total cholesterol; LDL, low-density lipoprotein; PCR, polymerase chain reaction.

Postoperatively, the patient was treated with 1% prednisolone acetate for a duration of 1.5 years. At the 18-months follow-up evaluation, the right eye graft remained clear, and the BCVA showed a modest improvement to 0.1. The residual scleral lesion remained stable without evidence of recurrence. The left eye is currently being managed with local corticosteroid eye drops and remains under close clinical surveillance.

A chronological summary of the patient’s clinical course, including historical events, diagnostic workup, and treatment outcomes, is presented in Table 2.

**TABLE 2:** Timeline of key clinical events and interventions.

Timeframe	Clinical event and management
20 years prior	Patient sustained bilateral metallic foreign body penetration trauma.
Past (date unknown)	Underwent partial excision of limbal lesions at another hospital (pathology reported as “benign”).
2 years prior	Bilateral corneoscleral lesions began to demonstrate marked progressive enlargement.
1 month prior	Superficial biopsy in outpatient clinic showed “chronic inflammation”; lesions showed no clinical response to topical corticosteroids.
Admission (Day 0)	Presented with severe visual impairment (OD: 0.02). AS-OCT revealed deep stromal invasion.
Surgery (Day 2)	Corneal lesion excision combined with large-diameter (13 mm) lamellar keratoplasty and intralesional injection of TA was performed on the right eye.
Post-op Day 2	Patient developed a minor interface hemorrhage in the right eye.
Post-op Day 3	Complication was successfully managed with anterior chamber/interface irrigation.
Post-op 18 months	Right eye graft remained clear, BCVA improved to 0.1, without residual tumor recurrence. Left eye condition remained stable under topical therapy.

## Discussion

Adult-onset xanthogranuloma (AOXG) involving the corneoscleral limbus represents an exceptionally rare, benign, reactive subtype of non-Langerhans cell histiocytosis (non-LCH). Current literature suggests that multiple limbal xanthogranulomas are extremely rare, whereas most reported cases manifest as solitary, unilateral nodules (Table 3) (8). Our case is particularly distinctive due to the unique combination of its prolonged 20-years disease course, bilateral symmetrical involvement, extensive invasion into the deep corneal stroma, and the successful application of AS-OCT-guided LKP combined with adjunctive corticosteroid therapy. Owing to these atypical clinical features, establishing a definitive diagnosis necessitated a rigorous multidisciplinary approach that incorporated the evaluation of ocular clinical mimics, exclusion of potentially fatal systemic histiocytic disorders, and modern molecular profiling.

**TABLE 3:** Summary of reported cases of adult-onset conjunctival and limbal xanthogranuloma in the literature.

References	Age/sex	Laterality and location	Clinical presentation	Treatment	Follow-up and outcome
Collum et al. (14)	17/M	Unilateral, limbus	Pink tumor	Excision, followed by superficial keratectomy and lamellar graft	4 years, no recurrence
Harvey et al. (15)	30/M	Unilateral, limbus	Yellow rapidly growing nodule	Lamellar dissection	4 months, no recurrence
Wang et al. (16)	45/M	Unilateral, limbus	Yellow-pink mass	Excisional biopsy	5 months, no recurrence
Kobayashi et al. (17)	40/F	Unilateral, limbus	Yellow-orange growth	Excision, followed by keratoplasty and subconjunctival TA	2 years, no recurrence
Mohamed et al. (18)	39/M	Unilateral, limbus	Yellowish nodule	Surgical excision	4 months, no recurrence
Hirata et al. (19)	48/F	Unilateral, limbus	Yellow-orange mass	Lamellar dissection	15 months, no recurrence
Hermel et al. (20)	35/M	Unilateral, limbus	Yellowish pink mass	Intralesional TA injections	6 months, no recurrence
Lee et al. (21)	43/M	Unilateral, bulbar conjunctiva	Two yellowish lumps	Conjunctivectomy and cryotherapy	2 months, no recurrence
Kontos et al. (22)	67/M	Unilateral, limbus	Yellowish dome-shaped mass	Excisional biopsy	7 months, no recurrence
Kim et al. (10, 23)	58/F	Unilateral, bulbar conjunctiva	Yellow-orange mass	Simple excision	12 months, no recurrence
Castro-Gómez et al. (24)	25/F	Unilateral, limbus	Yellow-orange elevated lesion	Simple surgical excision	5 years, no recurrence
Aggarwal et al. (25)	33/M	Bilateral, conjunctiva and limbus	Symmetrical yellow lesions	Excision + AMG (later systemic Tx for ECD)	20 months, ocular stable

M, male; F, female; TA, triamcinolone acetonide; AMG, amniotic membrane grafting; Tx, treatment; ECD, Erdheim-Chester disease.

Clinically, bilateral, fleshy, and highly vascularized corneoscleral masses must initially be differentiated from conditions such as ocular surface squamous neoplasia (OSSN), limbal dermoids, and lipid keratopathy (9). The 20-years indolent clinical course, combined with the absence of epithelial dysplasia, effectively excluded OSSN. Primary lipid keratopathy is generally characterized by an avascular presentation secondary to localized degenerative changes, whereas our patient’s lesions exhibited prominent feeder vessels and extensive cellular infiltration (10).

From a histopathological and immunohistochemical standpoint, the updated World Health Organization (WHO) classification of histiocytic disorders depends substantially on immunophenotypic characterization (11). The identification of Touton giant cells on H&E staining is considered a hallmark of the juvenile xanthogranuloma (JXG) family of disorders. More importantly, the IHC panel exhibited strong positivity for CD68 and CD163, together with complete negativity for CD1a, Langerin, and S-100. This distinctive immunophenotypic profile clearly differentiates AOXG from Langerhans cell histiocytosis (LCH), which characteristically exhibits expression of CD1a and Langerin. Furthermore, negative immunostaining for Melan-A and HMB-45 excluded amelanotic conjunctival melanoma from the differential diagnosis.

At systemic and molecular levels, confirmation of isolated AOXG requires careful differentiation from the broader and potentially life-threatening spectrum of adult xanthogranulomatous diseases (AXGD) involving the orbit and ocular adnexa. As defined by Sivak-Callcott et al., the AXGD spectrum encompasses AOXG, adult-onset asthma and periocular xanthogranuloma (AAPOX), and necrobiotic xanthogranuloma (NXG) (12). As detailed in our case presentation (Table 1), the absence of systemic manifestations, normal serum/radiological findings, and the absence of BRAF V600E mutation enabled definitive exclusion of these systemic mimics and supported the diagnosis of isolated bilateral AOXG.

Following the exclusion of systemic neoplastic, autoimmune, and genetically driven clonal etiologies, the pathogenesis underlying this extensive, localized lesion becomes particularly intriguing. We propose a speculative multifactorial hypothesis for this patient. We speculate that the initial pathogenic event may have been bilateral metallic foreign body trauma sustained 20 years earlier. This trauma likely disrupted the delicate limbal barrier, thereby inciting chronic, low-grade granulomatous inflammation and promoting localized neovascularization. The second proposed hit in this hypothesis is the patient’s underlying systemic hyperlipidemia. The presence of prominent conjunctival feeder vessels may have facilitated persistent extravasation of serum lipids into the chronically inflamed limbal microenvironment. These lipids were subsequently phagocytosed by local macrophages, leading to their transformation into foamy histiocytes and eventual fusion into Touton giant cells. Although there was no evidence of systemic histiocytosis, this synergistic interaction may have contributed to the massive, bilateral, and relentlessly progressive disease course observed in this patient.

Managing extensive limbal AOXG is inherently challenging. Surgical excision is indicated to prevent visual axis obscuration. In the present case, high-resolution AS-OCT played a pivotal role by accurately delineating the posterior extent of the lesion, thereby confirming deep stromal invasion without evidence of full-thickness corneal perforation (13). Consequently, a large-diameter LKP was carefully selected to restore and maintain a clear visual axis while ensuring essential tectonic stability. In addition, given the known corticosteroid responsiveness of non-LCH lesions, an intraoperative intralesional triamcinolone acetonide injection was administered into the residual vascularized scleral bed. This combined strategy of surgical debulking followed by targeted local pharmacologic therapy provided excellent anatomical control, although the functional visual recovery was ultimately modest due to the chronicity of the disease.

The primary limitation of this report is its inherent nature as a single case study, thereby limiting the generalizability of the findings. Additionally, although the 18-months follow-up demonstrated graft clarity and the absence of clinical recurrence, lesions within AOXG and JXG-spectrum may exhibit delayed local recurrences, particularly in cases where residual disease is intentionally preserved at the scleral margins to maintain globe integrity. Therefore, extended long-term follow-up data and future investigations involving larger patient cohorts are required to comprehensively validate the long-term safety and therapeutic efficacy of combining lamellar keratoplasty with adjunctive intralesional corticosteroid therapy. Finally, in relation to the CARE case-report framework, this report also has a limitation in that it lacks a direct patient perspective, as the patient was unavailable to provide a personal narrative at the time of publication.

## Conclusion

We present a rare case of bilateral, massive limbal adult-onset xanthogranuloma characterized by deep stromal invasion. Long-term chronic trauma and concurrent hyperlipidemia may be predisposing factors. Multimodal imaging, including AS-OCT, is crucial for evaluating the depth of invasion. For deep, vision-threatening lesions, LKP combined with intralesional corticosteroid injection may be an effective therapeutic modality in selected cases. Long-term follow-up is necessary due to the potential for local recurrence.

## Data Availability

The original contributions presented in this study are included in this article/supplementary material, further inquiries can be directed to the corresponding author.
